# Supplementary material for: Deficiency of Glucocerebrosidase Activity beyond Gaucher Disease: PSAP and LIMP-2 Dysfunctions
Source: Int J Mol Sci. 2024 Jun 16;25(12):6615. doi: 10.3390/ijms25126615 (PMC11204053; doi:10.3390/ijms25126615)
Supplement: Supplementary file 1 [file ijms-25-06615-s001.zip › ijms-2989914-supplementary.pdf]

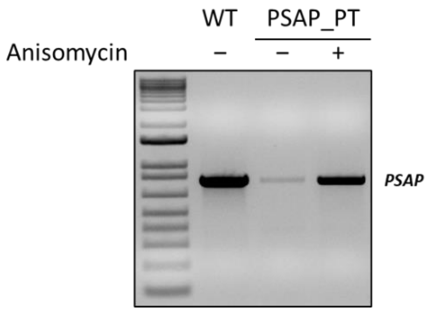

Supplementary Figure S1 | *PSAP* mRNA expression and nonsense-mediated decay in PSAP_PT.

Supplementary Table S1 | Genotypes, phenotypes, and GCase activity data of GD patients with biallelic pathogenic variants in *GBA1* gene included in this study. GenBank accession no. J03059.1. *GBA1* cDNA accession number NM_000157.3; protein accession number NP_000148.2. *Abbreviations: NA = non-available; ND = non-detected.*

**Patient**	***GBA1* Genotype**	**Predicted protein***		*GCase activity (nmol/mg/h)*			**Phenotype**
		**HGVS nomenclature**	***old nomenclature***	*Leucocytes nmol/mg/h*	*Fibroblasts nmol/mg/h*	*Plasma nmol/ml/h*	
GBA1_PT1	c.1448T>C / c.1448T>C	p.(L483P) / p.(L483P)	L444P / L444P	1.4	7.50	ND	GD3
GBA1_PT2	c.1448T>C / c.1448T>C	p.(L483P) / p.(L483P)	L444P / L444P	0.9	NA	ND	GD3
GBA1_PT3	c.1448T>C / c.1448T>C	p.(L483P) / p.(L483P)	L444P / L444P	NA	7.60	NA	GD3
GBA1_PT4	c.1448T>C / c.475C>T	p.(L483P) / p.(R159W)	L444P / R120W	1.1	NA	ND	GD2
GBA1_PT5	c.508C>T / c.680A>T	p.(R170C) / p.(N227I)	R131C / N188I	0.6	NA	ND	GD2
GBA1_PT6	c.1448T>C / c.508C>T	p.(L483P) / p.(R170C)	L444P / R131C	1.0	NA	ND	GD2
GBA1_PT7	c.508C>T / c.508C>T	p.(R170C) / p.(R170C)	R131C / R131C	NA	2.86	NA	GD2
GBA1_PT8	c.882T>G, c.1342G>C / c.882T>G, c.1342G>C	p.(H294Q), p.(D448H) / p.(H294Q), p.(D448H)	H255Q, D409H / H255Q, D409H	NA	1.73	NA	GD2

* GCase protein presents a signal sequence of 39 amino acidic residues, which are cleaved after directing the polypeptide to the ER. Historically, *GBA1* variants were numbered, indicating as amino acidic residue number 1 the first residue after the cleavage of the signal peptide. Even though this old nomenclature does not comply with the nomenclature standards of the Human Genome Variation Society (HGVS), it is still in use. Thus, we reported the predicted protein using both nomenclatures.

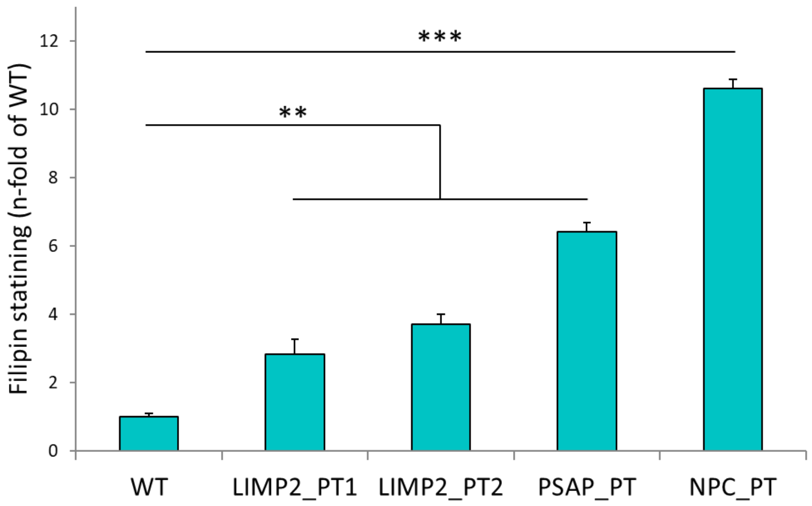

Supplementary Figure S2| Filipin staining quantitation. Results are expressed as mean ± SD of three independent experiments. ** p-value < 0.01; *** p-value < 0.001.

Supplementary Table S2 | *PSAP* primer sequences

Primer	Sequence (5'-3')
PSAP_1F	TCCCTTCCCTTTCTCCAAGT
PSAP_1R	CATTCTGGGGCAGATGGAC
PSAP_2F	TGTCCCATACAGCTTGGTGA
PSAP_2R	CTAAGGGGACCCAAGAGGAG
PSAP_3F	AGGGTGGAGAACCAGGATGT
PSAP_3R	TCAGGCCTACACCATTCCTC
PSAP_4F	TGTCTGCTTTTGCCTGATTG
PSAP_4R	TAACCCCAGGGCAAGTTACA
PSAP_5F	TTGGTATGCTGAGCAGTTTGA
PSAP_5R	GCCCCAGTTTAAGAACCACA
PSAP_6F	CTAATGCTGCAGCCCTGAGT
PSAP_6R	TCCAGATGCCATTAACCAAA
PSAP_7F	TTTGTTGGTTGGGTTTTGGT
PSAP_7R	CCCGTAACAGGGGAGGTACT
PSAP_8F	AGCATTTCCCCTGAACCTCT
PSAP_8R	AGATGAGACCCCCAAAACCT
PSAP_9F	AACAGACCAGGTGTCCTTGG
PSAP_9R	CAGGGAACCGAAAGAAACAA
PSAP_10F	TGGTCTCTGTGTCCCCTTTC
PSAP_10R	AGCCTAGAGGTCCCACTGGT
PSAP_11F	ACCATTGCCCTGCTTTGTAG
PSAP_12R	CAAAATGTACCCCAGCCTTG
PSAP_13F	GGAGCTCTCAGGGAACAGTG
PSAP_14R	AAAGCAGGGTGGAGAGTTGA
PSAP_15F	GATCCTGTCCTGGGTCTTCA
PSAP_15R	CAGAGAGAAAAGGGGCACTG
PSAP RNA F	GCTATGTACGCCCTCTTCCT
PSAP RNA R	CTCCTTGGGTTGCTGATCCT
